# A loss of consciousness in a teenage girl with anorexia nervosa, due to polydipsia: case report and a minireview

**DOI:** 10.1007/s40519-018-00636-x

**Published:** 2019-02-02

**Authors:** Aneta Krogulska, Dominika Nowicka, Zbigniew Nowicki, Monika Parzęcka, Agnieszka Sakson-Słomińska, Renata Kuczyńska

**Affiliations:** 1 Department of Pediatrics, Allergology and Gastroenterology, Collegium Medicum Bydgoszcz, Nicolaus Copernicus University of Toruń, M. Curie Skłodowskiej 9, 85-094 Bydgoszcz, Poland; 2 Student gastroenterological research group, Department of Pediatrics, Allergology and Gastroenterology, Collegium Medicum Bydgoszcz, Nicolaus Copernicus University of Toruń, Bydgoszcz, Poland; 3 Department of Pediatric Endoscopy and Gastrointestinal Function Testing, Collegium Medicum Bydgoszcz, Nicolaus Copernicus University of Toruń, Bydgoszcz, Poland

**Keywords:** Anorexia nervosa, Complications, Malnutrition, Polydipsia, Hyponatremia, Water intoxication

## Abstract

**Purpose:**

Anorexia nervosa is a chronic disease which may result in various complications. In pediatric clinical practice, it is common to observe complications related to progressive cachexia caused by malnutrition; however, cases of severe complications, like electrolyte disorders, which represent a direct threat to life, due to polydipsia, are rarely observed. The purpose of this study is to highlight that excessive drinking is of primary importance in anorexia nervosa patients, as it can result in severe medical complications, including increased risk of death.

**Methods:**

We report the case of a 13-year-old girl with anorexia nervosa, who was referred to hospital with seizures, disorders of consciousness, and cardiorespiratory failure.

**Results:**

The unstable condition of the patient was attributed to hyponatremia (119 mmol/l), decreased serum osmolality (248 mmol/kg), and decreased urine osmolality (95 mmol/kg) caused by polydipsia (water intoxication) and persistent vomiting. The presented girl was drinking large amounts of water prior to a weigh-in to falsify her low body weight.

**Conclusions:**

Polydipsia is a common problem reported by patients with eating disorders, but one which rarely leads to serious clinical complications, due to severe hyponatremia. This case underscores the importance of careful evaluation of fluid intake and the need for regular monitoring of serum electrolytes in patients with anorexia nervosa. All clinicians treating patients with such disease, as well as the parents of sick children, should be familiar with this life-threatening condition.

**Level IV:**

Evidence obtained from multiple time series with or without the intervention, such as case studies.

## Introduction

Anorexia nervosa (AN) is an eating disorder (ED) characterized by an intense fear of weight gain and a disturbed body image, which motivates severe dietary restriction or other weight-loss behaviors such as purging or excessive physical activity [1–3]. Low body weight or low body-mass index (BMI) is the central feature of AN [4]. There are two subtypes of AN: a restricting type and a binge-eating/purging type [5]. According to the DSM-5 criteria, to be diagnosed as having AN a person must display: (1) persistent restriction of energy intake leading to significantly low body weight (in context of what is minimally expected for age, sex, developmental trajectory, and physical health); (2) either an intense fear of gaining weight or of becoming fat, or persistent behavior that interferes with weight gain (even though significantly low weight); and (3) disturbance in the way one’s body weight or shape is experienced, undue influence of body shape and weight on self-evaluation, or persistent lack of recognition of the seriousness of the current low body weight. Amenorrhea no longer exists as criterion [3]. Patients with AN often develop weight concerns and subsequent behavioral change directed toward weight loss 6–12 months before the full clinical diagnosis [5].

AN can affect patients of all ages, sexes, races, and ethnic origins; however, adolescent girls and young adult women are particularly at risk [4]. The lifetime prevalence of AN is recorded between 1.2 and 2.2%, with an average point prevalence of 0.29% in 15–24-year-old females [6, 7]. It is suggested that the highest incidence rates for AN occur among females aged between 15 and 19 years. Incidence rates were found to rise with age [8]. Recent evidence suggests that the overall rates of AN have remained stable over the past few decades [8–10]. However, increasing trend, especially in younger age groups, especially girls were also reported [6, 11]. So far, it is unclear whether there is an actual increase of early onset AN or just an increase of utilization of health services by younger patients [6]. According to experts from The National Health Service, the number of admissions to hospital of patients with potentially life-threatening ED has almost doubled over the past 6 years and is the highest that they have been in at least a decade [12]. AN was the most frequent cause of hospitalization within all ED and suicide attempt-related hospitalizations were most common among patients with AN [9]. The incidence of AN differs, according to geographical location. The very low prevalence in Africa, Latin America and among Hispanics/Latinos in the USA compared with the rates in Western countries, but also compared to at least some Asian countries, such as China and Japan were reported [13].

AN can affect every organ system: cardiovascular, gastrointestinal, electrolytes, endocrine, bone, renal, hematologic, pulmonary, neurologic, and psychiatric and cause many medical complications [7]. Although most of the medical complications of AN are treatable and reversible with optimal medical care and restoration of a healthy weight, it is considered that some of them may have permanent adverse effects. The earlier they are diagnosed and treated, the better are the long-term chances of full recovery [14]. In children and adolescents with longstanding AN, some clinical abnormalities may be irreversible, including growth impairment, structural brain changes, decreased bone density, and infertility [15]. Recently, long-term cognitive acumen dysfunction and emotional dysfunction are considered as a result of AN [14].

Outcomes differ across age groups, with higher rates of a better outcome, full recovery, and lower mortality in adolescents than in adults [3, 16]. At least 40% of patients with AN (and more in children), who are treated, will make a full recovery [16]. Long-term follow-up studies have confirmed two broad outcome groups with either a good outcome or chronic course with a high risk of death [4, 16].

AN has the highest mortality rate of any psychiatric disorder [6, 7, 13, 14, 17, 18]. The mean mortality in adolescents is 2% and in adults 5% [16]. Many of the deaths are attributable to medical complications which are a direct result of weight loss and malnutrition [14, 17]. According to Smink et al., most deaths due to AN are particularly a consequence of cardiac complications and severe infections [6]. Arcelus et al. showed that death is most often secondary to medical complications of starvation (50%) or suicide (50%) [19]. Based on a large prospective clinical longitudinal study, Fichter et al. shown that physiological effects of starvation and purging behaviors were responsible for over half of deaths resulting from medical complications of AN [17]. A form of “purging behavior” used to eliminate calories with diuresis is polydipsia. Polydipsia is defined as the consumption of excessive amounts of fluid, larger than normal volumes (4–20 l daily) [20].

In pediatric clinical practice, it is common to observe complications related to progressive cachexia caused by malnutrition; however, cases of severe, acute complications, like electrolyte disorders, which represent a direct threat to life, are rarely observed. The following report describes the case of a patient with these complications.

## Case presentation

A 13-year-old girl was admitted to the Emergency Room (ER) with disorders of consciousness: she was incapable of verbal communication, unable to follow commands, disorientated, and demonstrated balance disorders. Due to the fast progress of the symptoms, including loss of consciousness, she was referred to the Pediatric Intensive Care Unit (PICU). Her prior medical history revealed that she had experienced persistent vomiting and had complained of a headache 2 days before hospital admission. Parents denied the use of medicines, psychotropic agents, drugs or trauma. They reported that their daughter had been on a weight-loss diet for few months and she had not menstruated for 6 months.

The patient remained in the PICU in a critical condition: unconscious, with Glasgow Coma Scale (GCS) of 3. She demonstrated a normal reaction for pain stimulus, slow pupil response to light, decreased subcutaneous tissue, regular heart rate of 50 beats per minute, respirations of 22 per minute, blood pressure of 120/65 mmHg, oxygen saturation 92%, and temperature of 37.2 °C. Laboratory testing revealed electrolyte imbalance (Table 1).

**Table 1:** Laboratory results at admission to PICU and at the 8th day

Serum concentration	At admission	At 8th day	Reference range
Na (mmol/l)	119	136.5	135–145
Cl (mmol/l)	88	102.3	98–106
P (mmol/l)	0.85	1.12	1.3–2.26
K (mmol/l)	3.6	4.1	3.5–5.5
CRP (mg/l)	0.2	0.25	< 0.5
WBC (mm^3^)	5100	4740	4000–12,000
Creatinine (umol/l)	59	54	53–106
Glucose (mmol/l)	5.05	5.0	3.33–5.56
BUN (mg/dl)	10.0	14.3	5.6–18.8
Plasma osmolality (mmol/kg H_2_0)	248	283	270–300
Urine osmolality (mmol/kg H_2_0)	95	371	250–1300

Shortly, after admission to PICU, generalized tonic–clonic seizures were reported. The patient was sedated and intubated. Antibiotic therapy (cefuroxime), antiviral treatment (acyclovir), antioedematous treatment (mannitol), total parenteral nutrition, and hypertonic saline infusion were administered. She was given 150 mL of 3% NaCl in bolus which brought her sodium to 125 mmol/l. In response to persistent hyponatremia, an infusion of concentrated NaCl was continued.

**Fig. 1:**
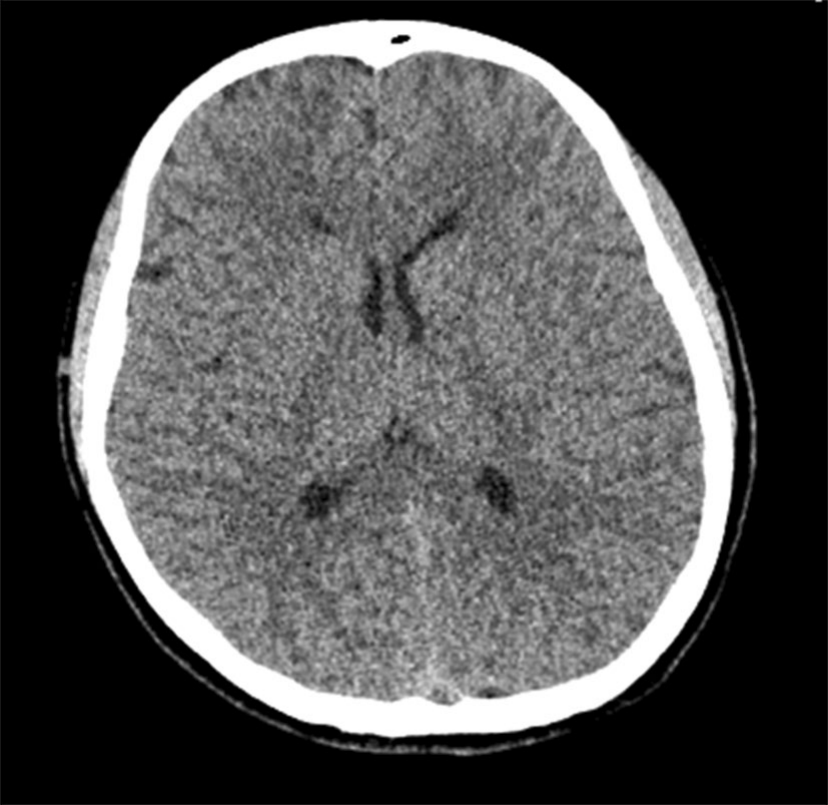
Patient’s brain computed tomography: no visible abnormality

A differential diagnosis was performed. A computed tomography (CT) of the head was performed, which demonstrated no evidence of acute trauma or cerebral edema (Fig. 1). Negative toxicology tests excluded poisoning. Cerebrospinal fluid (CSF) analysis excluded neuroinfection. MRI scan of the head revealed no abnormalities.

Over the following days, polyuria and hypotension were observed. The catecholamines were applied and antioedematous treatment was discontinued. Clinical improvement was observed on day 3, so analgosedation and the catecholamine infusions were discontinued. On day 5, the patient was extubated. On day 7, the patient demonstrated balanced electrolyte levels and cardiorespiratory competence, and so was referred to the Department of Pediatrics. Subsequently, periodical polyuria was observed. Her general condition was good, but adynamic. Physical examination revealed conjunctival petechiae and thin subcutaneous tissue; body weight was 45 kg (25c), body height 170 cm (97c), and BMI was 15.57 (3c). Current results are presented in Table 1. Hormonal disorders and renal damage were taken into consideration for the differential diagnosis of hyponatremia: the tests results were within reference values.

After acute management, further past history was taken from the patient’s parents, and it was revealed that due to observed weight loss, they decided that the girl should maintain minimum body weight of 50 kg. Under this condition, they would allow her to continue her dance training (this is her passion). For the past few weeks, they were checking her body weight weekly. It was found that she had been drinking a large amount of water (about 4 l) before being weighed, and had done so the day before hospitalization.

During her stay in hospital, her condition improved, serum sodium stabilized at 140 mmol/l, and at discharge, she was referred for psychiatric evaluation. The final diagnosis was the severe acute, hypotonic hyponatremia associated with polydipsia during the course of AN.

## Discussion

This study describes a rare case of severe and life-threatening hyponatremia in a girl with AN. Water intoxication caused by polydipsia resulted in ion disturbances. The syndrome of neuropsychiatric symptoms with severe hyponatremia is a complication of polydipsia-induced water intoxication that is rarely seen in patients with AN. In fact, very few cases of polydipsia among patients with AN have been reported in the literature [21, 22].

AN generally starts with dieting behavior that may evoke no concern [23]. The clinical symptoms of it are not usually overt, and so may be underemphasised or unnoticed by parents and general practitioners for a long period. Many individuals have poor insight into their condition and do not consider themselves to be ill. Patients with AN typically consult physicians for secondary amenorrhoea or significant body weight loss in a short period of time [23]. The condition of the presented patient was so well hidden that the parents had not sought medical help, despite the amenorrhoea occured. She was diagnosed with AN during hospitalization only after psychiatric evaluation.

AN has been recognised as an ED with one of the highest risks of a fatal outcome [24]. In the course of the disease, numerous organ complications may develop, depending on the disease stage and severity of cachexia [14]. Our case was not related to progressive cachexia, but it is an example of acute, rare, life-threatening complication due to polydipsia.

Psychogenic polydipsia (PPD), also called primary polydipsia or compulsive polydipsia, is a clinical disorder characterized by excessive water drinking in the absence of a physiologic stimulus to drink or any underlying organic disease process. It occurs in up 25% of psychiatric patients. Although psychogenic polydipsia is relatively common in psychiatric population, only one-fifth to one-third of polydipsic patients will experience symptomatic hyponatremia, a potentially life-threatening complication [25]. PPD most commonly occurs in schizophrenic patients (80%), but other disorders including bipolar disorder, chronic alcoholism, mental retardation, autism, severe behavioral disorder, dementia, and AN, like in our case, are also associated. A condition of water and electrolyte imbalance in patients with psychosis was named PIP-syndrome (psychosis–intermittent hyponatremia–polydipsia). According to Santonastaso et al., polydipsia is often reported by patients with ED, but it is rarely associated with severe health consequences [26]. Abraham et al. note that only 17% of patients with ED consumed an appropriate amount of fluids, and consumption in the whole group ranged from 250 mL to even 6 l per day [27].

PPD has multifactorial pathogenesis, involving dysfunctional hypothalamic thirst regulation. A lower thirst threshold is connected with hyperactivity of hypothalamic thirst regulation centers and hippocampal dysregulation, which leads to compulsive drinking, thereby decreasing the tonicity of blood [22, 28]. Another theories contribute to cause polydipsia are: dysregulation of antidiuretic hormone (ADH) and increased affinity of opioid agonists to their receptors in hyponatremic environment in polydipsic patients [22, 28]. Inhibition of ADH leads to significant renal excretion of fluids and thus prevents blood from becoming too dilute, perpetuating a cycle of polydipsia and polyuria. However, as patients continue to have extreme thirst, they eventually drink beyond the kidneys’ capacity to dilute urine, resulting in hyponatremia [22]. Antipsychotics have also been speculated as the cause for the onset or worsening of polydipsia [28].

Although the background of polydipsia in patients with AN may vary, the most common reasons include a willingness to suppress hunger and eliminate toxins from the body. Water may also be drunk to induce vomiting and satisfy physiological needs, such as thirst, following physiological effort. One of the other important reasons is a wish desire to cheat on weight measurements. Polydipsia may be also interpreted as a form of bulimia in the spectrum of ED [18, 22].

Usually, polydipsia leads to mild hyponatremia, rarely severe ones resulting in fatal neurologic consequences. Such severe complications are seldom observed in patients with AN. Hyponatremia is seldom caused alone by polydipsia. Various pathomechanisms in anorexia patient’s leading to fluid imbalanced were noticed: releasing of antidiuretic hormone and osmoregulation disorders; deficiency in cell volume regulatory mechanisms and abate glomerular filtration rate [29]. These disorders may also contribute to the development of electrolyte disturbances.

Water intoxication is a rare condition that originates from polydipsia, with a potentially fatal outcome, due to hyponatremia. In four cases of polydipsia with water intoxication, presented by Radojevic et al., there is a combination of polydipsia, impaired free water excretion, and increased release of antidiuretic hormone [30].

Hyponatremia occurs when plasma sodium is less than 135 or 130 mmol/l. It may be caused by primary sodium or potassium deficits, resulting in water excess or combination all of these conditions. Mostly, hyponatremia is induced by higher water level due to the sodium and potassium imbalance [31]. In relation with plasma osmolality hyponatremia might be classified as isotonic, hypertonic or hypotonic. Due to the fact that sodium is the prevailing extracellular osmole, hyponatremia mostly is hypotonic and it may be categorized on the grounds of volume status as normo or hypo/hypervolemic hypotonic hyponatremia [31]. Hypotonic hyponatremia is the most frequent form of hyponatremia and occurs when water intake exceeds the water expelling amount. Deterioration of renal water excretion is the main cause of hypotonic hyponatremia. In some cases, however, it may be caused by excessive water intake with normal or almost normal functioning kidney. All these cases are included in PPD.

The symptoms of hyponatremia may include nausea, vomiting, headache, confusion, somnolence, seizures and coma, as well as cardiorespiratory failure, all of which were observed in our case. According to symptoms classification, the girl was diagnosed with severe hyponatremia, similar to evaluation based on serum sodium concentration (Table 2) [20, 32].

**Table 2:** Classification of hyponatremia [20]

Severity	Na serum concentration (mmol/l)	Symptoms
Mild	135–130	Usually asymptomatic
Moderate	129–125	Nausea without vomiting
		Confusion
		Headache
Severe	< 125	Vomiting
		Cardiorespiratory failure
		Abnormal and deep drowsiness
		Convulsions
		Coma (≤ 8 GCS scale)

Following water intoxication, brain oedema develops due to to a very fast decline of the sodium level in the serum. Even a small decrease (< 125 mEq/l) induced by superabundant consumption of plain water or hypotonic fluids causes impermanent water transfer into cells from the extracellular ambience to sustain adequate osmolarity. This process leads to brain swelling which is followed by abnormal neurological symptoms, including seizures. Young age and female hormones are the major risk factors for the development of hyponatremic encephalopathy.

Hyponatremia treatment is based on symptoms, the endurance of hyponatremia and the patient’s volume level. The possibilities of developing myelinolysis in patients with severe hyponatremia are very seldom, because the sodium level can be corrected quickly at the rate up to 2 mEq/l/h. In the case of chronic hyponatremia, it is suggested slower rate of infusion, no more than 8 mEq/l/day [20].

The data presented in publications and from this case indicate that the amount of fluids consumed by patients with AN have to be monitored, both at hospital and at home. In patients suspected of consuming greater amounts of fluids, periodical tests for serum sodium levels are necessary to detect hyponatremia as soon as possible, and to prevent its life-threatening consequences. Patients should also be given psychoeducation to make them more aware of the possible dangers of excessive water intake. It is worth emphasising that the treatment used in this child, before hospitalization, was not appropriate: for a few months, the child had been “treated” only by her father, a dietician, who had been trying to track her body weight, but had not been monitoring the amount of fluids consumed or the results of laboratory tests.

Treatment of AN is based mainly on nutritional rehabilitation and specialized psychotherapy programme [1, 2, 5, 24]. Although food is considered the best medicine, nutritional counselling alone is not an adequate treatment [4]. It is suggested that the use of nasogastric tube feeding is more efficient than other approaches in promoting weight gain [33]. Although enteral nutrition is an essential life-saving treatment in severe cases of AN, it does not guarantee long-term success or recovery [34]. The outpatient psychosocial interventions like: family and individual therapies are the initial treatment of choice for children and adolescents [1, 2, 5].The strong evidence within psychological treatment has family-based treatment (FBT) and Maudsley family therapy (MFT) [1, 2, 4]. Medication should not be used as the sole or primary treatment, but should be reserved for comorbid conditions and refractory cases [5]. According to American Psychiatric Association, most patients with AN can be treated as outpatients. Hospitalization is needed for those with more severe illness and those who do not improve with outpatient care [3].

## Conclusion

The monitoring of weight is insufficient to prevent negative sequelae or treat the underlying psychopathology in patients with anorexia nervosa. Severe hyponatremia is a rare, but serious complication in patients with psychogenic polydipsia, which can be observed in anorexia nervosa. All clinicians treating patients with AN, as well as the parents of sick children should be familiar with this life-threatening condition. We believe that this case report serves as a reminder of an important clinical lesson.
